# Quality of Life of Sudanese Children with Type One Diabetes in Sudan 2022: A Cross-Sectional Study

**DOI:** 10.1192/bjo.2025.10144

**Published:** 2025-06-20

**Authors:** Tebyan Ali, Danya Ibrahim, Moez Mohammed, Marfa Ali, Ahmed Hussien

**Affiliations:** Khartoum University, Faculty of Medicine, Khartoum, Sudan

## Abstract

**Aims:** This study aimed to assess the different dimensions of quality of life in children with type one diabetes including physical, social, emotional, and school function. Also, to identify the relationship between the quality of life and demography, duration of illness, and control of diabetes

**Methods:** The participants were selected randomly from the biggest two paediatric hospitals in Khartoum. The child and their parent or legal guardian were interviewed face-to-face. Descriptive statistics were employed to evaluate quality of life (QoL) using the Short Form-12 scale (SF-12). The Pearson correlation test was utilized to examine the relationship between QoL and various independent variables, including demographic factors (age, gender, parents’ education, residency, and occupation of both parents), family income, duration of illness, and diabetes control.

**Results:** Most participants were aged 13–18 years (63.8%), with 55.1% identifying as female. Most fathers had completed secondary education (36.2%), while a similar percentage of mothers had completed primary education (36.2%). Additionally, a significant proportion of fathers were self-employed (69.6%), whereas most mothers were unemployed (66.7%). Family income was reported to be less than 50,000 per month for 46.6% of families. A significant association was identified between QoL scores and gender, father’s occupation, and family income (p<0.005). Lower QoL scores were prevalent among 61% of participants. A notable correlation was also found between QoL and the promotion of exercise (p=0.002).

**Conclusion:** The study highlights a reduction in the social and emotional functioning of health-related quality of life (HrQoL) among children and adolescents. It concludes that the father’s occupation and monthly income are significant predictors of improved HrQoL, alongside exercise promotion. Additionally, female gender emerged as a predictor of lower HrQoL scores.

